# Integrative taxonomy using the plant core DNA barcodes in Sumatra's Burseraceae

**DOI:** 10.1002/ece3.9935

**Published:** 2023-04-07

**Authors:** Daniel M. Teklemariam, Oliver Gailing, Iskandar Z. Siregar, Fitri Yola Amandita, Carina C. M. Moura

**Affiliations:** ^1^ Department of Forest Genetics and Forest Tree Breeding University of Göttingen 37077 Göttingen Germany; ^2^ Centre of Biodiversity and Sustainable Land Use University of Göttingen Göttingen Germany; ^3^ Department of Silviculture, Faculty of Forestry IPB University Bogor Indonesia

**Keywords:** barcode gap, genetic distance, *matK*, species identification, tropical biodiversity

## Abstract

The high diversity and limited floral information in tropical forests often pose a challenge for species identification. However, over the past decade, DNA barcoding has been employed in tropical forests, including Sumatran forests, to enhance floristic surveys. This technique facilitates the discrimination of morphologically similar species and addresses the limitations of conventional species identification, which relies on short‐lived reproductive structures. This study aimed to evaluate the efficiency of *matK*, *rbcL*, and the combination of both chloroplast markers for species identification in Burseraceae by employing genetic distance and species tree inference. In this study, we collected 197 specimens representing 20 species from five genera of Burseraceae. The highest percentage of specimens' identification (36%) at the species level was obtained using *matK + rbcL*, followed by *matK* (31%), and *rbcL* (7%). The *matK* dataset presented the highest interspecific divergence with a mean of 0.008. In addition, a lack of barcode gap was observed in both markers, suggesting potential limitations of the core barcodes for distinguishing Sumatran species within Burseraceae. The monophyly test confirmed five species as monophyletic using Bayesian species tree inferences for *matK*. Overall, our results demonstrate that *matK* outperforms *rbcL* in species identification of Burseraceae, whereas their combination did not enhance species delimitation. To improve the molecular species assignments of this family, future studies may consider including more DNA markers in conjuction with *matK*, and broadening the availability of reference sequences for species that have not yet been included in the databases. The outcomes of molecular species identification vary depending on the taxonomic group under investigation. Implementation of phylogenomics for species delimitation and diagnostic marker development is strongly recommended for tropical biodiversity assessments, especially for poorly studied clades.

## Graphical abstract

Species identification in tropical forests is often difficult due to its high diversity and limited floral information, therefore DNA barcoding has been applied in tropical regions such as Sumatran forests to boost floristic surveys. In this study, we sequenced 197 specimens representing 20 species from five genera of the family Burseraceae using the barcode markers *matK* and *rbcL*. Our results showed that *matK* discriminated among 97% of the sequence pairs and identified a modest number of species from the Sumatran tropical rainforest. This reinforces the suitability of this marker for molecular species surveys of Sumatran Burseraceae species.

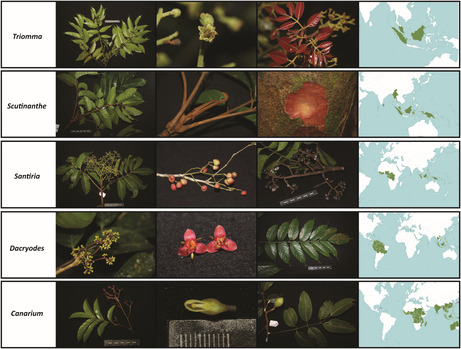

## 1 INTRODUCTION

DNA barcoding is the use of a short gene or amplicon sequence from a specific region of the genome which can be used to determine and differentiate species and to assign an unidentified sequence of individuals to species (Newmaster et al., 2006). An ideal barcode must show high interspecific genetic divergence to discriminate one species from another and have a low intraspecific genetic variation (Lahaye et al., 2008). In addition, it should be short enough to be recovered from degraded tissue such as highly processed materials or forensic samples (Chase et al., 2007). As a principle, this method utilizes the genetic variation among species to distinguish organisms (Hebert et al., 2003), in which a sequence of an unidentified specimen is compared to a sequence database of identified sequences (Stech et al., 2013).

Extensive studies have been carried out in the field of DNA barcoding, to be used for accurate and effective species identification (e.g., Amandita et al., 2019; Gonzalez et al., 2009; Hebert et al., 2003; Kress et al., 2009). The technique can be used to identify species that are difficult to distinguish based on their morphology and as a supporting tool in the classification and description of cryptic plant species (Hartvig et al., 2015). Due to the dwindling number of taxonomists and herbaria, the conventional taxonomy is insufficient to deal with the increasing demand for accurate and accessible taxonomic information (Newmaster et al., 2009). Thus, DNA barcoding could be a supplementary tool for species inventories and the conservation of biodiversity in areas with high diversity and limited floral information (Hartvig et al., 2015).

The *cytochrome oxidase 1* (COI) gene of the mitochondrial DNA is identified as a universal DNA barcode for animals. However, due to the low variation of nucleotides in the mitochondrial DNA of plants, COI is found inefficient for plants to be used as a universal barcode (Hollingsworth et al., 2011). Several studies suggested different marker genes as potential DNA barcodes for plants, for instance, *rbcL* (Chase et al., 2005; Kress & Erickson, 2007), *trnH‐psbA* (Kress et al., 2009), *matK* (Lahaye et al., 2008), *trnL* (Taberlet et al., 2007), and ITS (Chen et al., 2010; Kress et al., 2005).

Barcoding studies in plants have suggested discriminatory power by using *matK* and *rbcL* regions; however, these plastid genes failed to provide a barcode gap in many plant families, which means a clear separation between the genetic variation within and between species, allowing for the adequate differentiation between species using barcode markers (Vijayan & Tsou, 2010). These regions have ca. 70% species discriminatory power in plants (Vijayan & Tsou, 2010); despite their limitations, they are largely accepted as an integrated tool for plant identification together with morphological taxonomy.

Although the universal *matK* primers have been criticized for their low success rate in amplification (e.g., Kress & Erickson, 2007), on one hand, several researchers have suggested *matK* as sufficient DNA barcode in plants for its species‐level identification power (e.g., Hollingsworth et al., 2009; Lahaye et al., 2008). On the other hand, despite its easy amplification, sequencing, and alignment, *rbcL* has moderate identification power in most land plants (Hollingsworth et al., 2011). To overcome the limitations of universality, sequence quality, discriminatory power, the CBOL Plant Working group (2009) proposed the use of *matK* and *rbcL,* and their combination (*matK* + *rbcL*).

DNA barcoding has been increasingly applied during the last decade, especially to facilitate biodiversity studies of hyper‐diverse but taxonomically poorly known regions, such as Sumatran tropical rainforests (Amandita et al., 2019; Moura et al., 2019). Sumatra has been one of the largest tropical lowland forest areas with tree species diversity as high as ca. 10,600 (Roos et al., 2004). However, Sumatra experienced the highest deforestation rates within insular Southeast Asia between 2000 and 2010, with yearly deforestation rates above 5.0%, and its eastern lowlands represented extreme concentration areas of forest loss (Miettinen et al., 2011). The main drivers of land‐use changes and deforestation in this region are the rubber, pulp and paper, timber, and oil palm industries (Laumonier et al., 2010). Indonesia is a megadiverse country and ranks fifth on the list of the world's richest countries in terms of biological diversity (Pitopang et al., 2004). Since accurate identification of plant species and understanding of their phylogenetic relationships are the fundamental steps for conservation and sustainable utilization of plant resources (Kim et al., 2014), the application of DNA barcoding could be a supporting tool for conservation and biodiversity assessments in Sumatran forests.

In this study, DNA barcoding is applied to Burseraceae family in Sumatra to evaluate the efficiency of the barcodes for molecular species identification by employing species‐tree inferences and testing monophyly of the recovered clades. The coding plastid regions *rbcL*, *matK*, and their combination (*matk + rbcL*) are used, as recommended by the CBOL Plant Working group (2009), as the core barcodes for land plants. Species‐tree‐based barcoding methods are employed to increase the statistical power for sequence assignment where genetic distance is low and a barcode gap is almost absent (Mallo & Posada, 2016).

Burseraceae is a family of trees and shrubs. The species from this family are sometimes rupicolous inhabiting rocky terrains, very rarely scandent or epiphytic, with ca. 700 species in 18 genera divided into three tribes (*Canarieae*, *Protieae*, and *Bursereae*; Weeks et al., 2005). The family is close ally of Anacardiaceae, Rutaceae, Simaroubaceae, and Meliaceae (Soltis et al., 2000). It is well known for its fragrant resins, such as frankincense, myrrh, and copal which have great economic, medicinal, and cultural values (Langenheim, 2004).

In the current study, the general objective is to test DNA barcodes for species delimitation of Burseraceae from Sumatra. Specifically, we aimed to evaluate the efficiency of *matK*, *rbcL*, and the combination of both chloroplast markers for species identification by employing pairwise genetic distances and species‐tree inference.

## 2 MATERIALS AND METHODS

### 2.1 Study site

This study was carried out in two landscapes of Jambi Province, Sumatra, Indonesia: Bukit Duabelas National Park and Harapan Rainforest. The lowlands of Jambi have a tropical humid climate with two peak rainy seasons around March and December, with a dryer period during July and August. The region has an average annual temperature of 26.7°C and mean annual precipitation of approximately 2235 mm. The study area consists of natural vegetation, which is dominated by dipterocarps; however, due to logging concessions and forest conversion into agricultural land, the lowlands of Jambi Province experienced rapid large‐scale deforestation (Rembold et al., 2017).

### 2.2 Sample collection and morphological taxa identification

Samples were collected in 32 plots (50 m × 50 m) distributed on four land‐use types; logged‐over primary rain forest, jungle rubber agroforestry, rubber plantations, and oil palm plantations. Trees with >10 cm DBH were sampled in all plots. Leaf tissue of three specimens of each Burseraceae‐identified species was collected and dried in silica gel for further analyses. Herbarium vouchers of each species were prepared and stored at Indonesian herbaria (Herbarium Bogoriensis and BIOTROP Herbarium), and high‐quality photographs were taken for further identification.

All taxa were morphologically identified during the field inventory. Associated taxonomists classified each collected specimen to the species level by matching the herbarium vouchers with the reference vouchers from the Indonesian herbaria. Vouchers' IDs correspond to the sample IDs of this study (Table S1). The morphologically identified species were then later compared with the DNA barcode identification.

### 2.3 DNA extraction, PCR amplification, and sequencing

Tissue samples for DNA analyses of each morphologically identified species were used in this study. DNA extraction was then carried out for each specimen using the DNeasy 96 Plant Kit (Qiagen), following the manufacturer's protocol. DNA concentration was checked using 1% agarose gel electrophoresis with 1× TAE buffer solution and 4 μL Roti‐Safe dye.

The two plastid markers *matK* and *rbcL* were amplified from each extracted DNA sample using universal primers as listed in Table 1. For *matK*, we used the primer combination 3F_KIM_f and 3F_KIM_r. In case the amplification failed with the mentioned primer pairs, a second amplification was undertaken using the second primer pairs (390f and 990r). Amplification was achieved in 14 μL reaction mixture containing 1 μL diluted DNA sample, 1.5 μL PCR buffer (with 0.8 M Tris–HCl, 0.2 M (NH_4_)_2_SO_4_), 1.5 μL MgCl_2_ (25 mM), 1 μL dNTPs (2.5 mM of each dNTP), 1 μL of forward primer, 1 μL reverse primer (5 pM/μL each), 0.2 μL (5 U/μL) HOT FIREPol® Taq‐Polymerase (Solis BioDyne), and 6.8 μL ddH_2_O. PCR was performed using a Peltier Thermal Cycler Biometra (Analytic Jena). The thermal cycling was carried out with initial denaturation at 95°C for 15 min, followed by 35 cycles of denaturation at 94°C for 1 min, annealing at 50°C for 1 min, elongation at 72°C for 1.5 min, and ended with a final extension of 20 min at 72°C. All PCR products were verified prior to sequencing using 1% agarose gels and then excised from the gel and purified according to the innuPREP Gel Extraction Kit protocol (Analytic Jena).

**TABLE 1:** List of primers of *matK* and *rbcL* barcode regions used for amplification and sequencing of Burseraceae samples in this study.

Marker	Primer	Primer sequences (5′–3′)	References
*matK*	*3F_KIM_f*	CGTACAGTACTTTTGTGTTTACGAG	CBOL (2009)
	*1R_KIM_r*	ACCCAGTCCATCTGGAAATCTTGGC	CBOL (2009)
	*390f*	CGATCTATTCATTCAATATTTC	Schmitz‐Linneweber et al. (2001), CBOL (2009)
	*990r*	GGACAATGATCCAATCAAGGC	Gamage et al. (2006)
*rbcL*	*rbcL_f*	ATGTCACCACAAACAGAGACTAAGC	Kress and Erickson (2007)
	*rbcL_r*	GAAACGGTCTCTCCAACGCAT	Fazekas et al. (2008)

Each marker was prepared for bidirectional sequencing using the BrilliantDye v3.1 Terminator Cycle Sequencing Kit optimized for Dye Set Z (NIMAGEN). The sequencing reaction mixture contained 2 μL DNA template (5–10 ng), 4.5 μL ddH_2_O, 0.5 μL BrilliantDye v3.1, 2 μL 5× Sequencing buffer, 1 μL forward or reverse primer (5 pM/μL; Table 1). The sequencing cycle included: initial denaturation at 96°C (1 min) followed by 35 cycles of 96°C (10 s), 45°C (10 s), and 60°C (4 min) with a final extension period of 20 min at 72°C. Subsequently, the samples were purified with DyeEx® 96 Kit (Qiagen) following the manufacturer's protocol. Finally, the obtained sequences were analyzed using an ABI Prism Genetic Analyzer 3130xl with the Sequence Analysis software v5.3.1 (Applied Biosystems).

### 2.4 DNA sequence analysis

The complementary bidirectional DNA sequences from each sample were trimmed on both sides if appliable and assembled using CodonCode aligner software (https://www.codoncode.com/aligner/n.d.). Each assembled contig was manually checked for sequencing errors and edited where needed. Subsequently, the generated fasta files of consensus sequences were aligned using the multiple sequences alignment algorithm Muscle in CodonCode aligner. Two locus DNA barcodes were concatenated using the aligned sequences of *rbcL* and *matK* in BioEdit Sequence Alignment Editor Software (Hall, 1999). Moreover, the C + G content was calculated in DnaSP v6 (Rozas et al., 2017), and the percentage of variable sites and Parsimony‐informative sites were assessed in MEGA 7 (Kumar et al., 2016).

Using BLAST algorithm, the best match for the generated sequences was searched in the National Center for Biotechnology Information (NCBI) nucleotide database. Following Amandita et al. (2019), the match between molecular and morphological classification was categorized into three levels: species, genus, and family. A sample is considered correctly identified at species level, when both morphological and molecular identifications match for its species name, whereas genus or family identification is considered to be correct, if both identifications match at genus or family level. If only one of the markers matches with the morphological identification, the assignment was counted as uninformative. In addition to that, based on the interspecific sequence divergence of the single markers and their combination, the number of species that can be discriminated was calculated and included in the genetic distance and phylogenetic analyses. The availability of sequences for the regions *matK*, *rbcL*, and other common plant barcodes (*trnH‐psbA* and *trnL‐F*) was verified in the NCBI database for the species analyzed in this study (Table S2). A limitation for DNA barcode assignments of tropical species is the availability of reference barcode sequences for the taxa investigated (Halmschlag et al., 2022; Moura et al., 2022; Wati et al., 2022).

Furthermore, to provide a more complete coverage of the family Burseraceae and to facilitate the phylogenetic placement of the barcode sequences obtained in this study, sequences of missing genera were downloaded from NCBI and aligned with the dataset to increase the number of species and genera included in the species‐tree inference, and species from family Anacardiaceae were included as outgroup (Table S3).

### 2.5 Genetic distances

The inter‐ and intraspecific genetic distances between the sequences of the sampled species were calculated using MEGA 7 (Kumar et al., 2016). The genetic distance is estimated by the proportion (*p*) of pairwise sequence nucleotide differences (*n*_d_) per site divided by the total number of nucleotides compared (*n*), as described below:

$$P=\frac{n_{d}}{n}$$

Wilcoxon rank‐sum test was used to calculate the significance of differences of the interspecific and intraspecific genetic divergence following Lahaye et al. (2008). To check if an overlap between inter‐ and intraspecific divergences is present, the frequency distribution of both genetic variations of each marker and the combined markers (*matK + rbcL*) was illustrated in box plots using R package ggplot2 (Wickham, 2011). The accuracy of a barcode to identify and delimit species depends on the existence of a gap between inter‐ and intraspecific genetic distances which is the so‐called barcode gap (Meyer & Paulay, 2005). For each barcode, genetic distances were calculated based on the best‐fit models in MEGA 7 (Kumar et al., 2016). DNA barcodes can be used to recognize flawed morphology‐based identification in the instance a barcode gap exists. This may vary according to the level of polymorphism of the barcode markers of the target organism.

### 2.6 Phylogenetic tree reconstruction and monophyly test

Phylogenetic trees were reconstructed using the aligned sequences with the Bayesian inference (BI) method. The Bayesian inference approach was applied in BEAST1.8.0 (Drummond et al., 2012) using Hasegawa, Kishino, and Yano (HKY) as a nucleotide substitution model for nucleotide sites and Yule model of branching, and it was run for 10^6^ generations through the CIPRES supercomputer cluster (Miller et al., 2010). Furthermore, by using Tree Annotator 1.8.0 (Drummond et al., 2012), a maximum clade credibility tree was generated. Finally, the trees generated were visualized using ITOL (Letunic & Bork, 2021). To test for monophyly of the clades at different taxonomic levels (species, genus, subtribe, tribe, and family), we used the package MonoPhy (Schwery & O'Meara, 2016) in R 4.2.2 (R Core Team, 2022). Post hoc ‘molecular’ identifications were confirmed afterward by taxonomists with access to the specimens in order to integrate both molecular and morphological identifications.

## 3 RESULTS

### 3.1 Sequencing success rate and characteristics of markers

**FIGURE 1:**
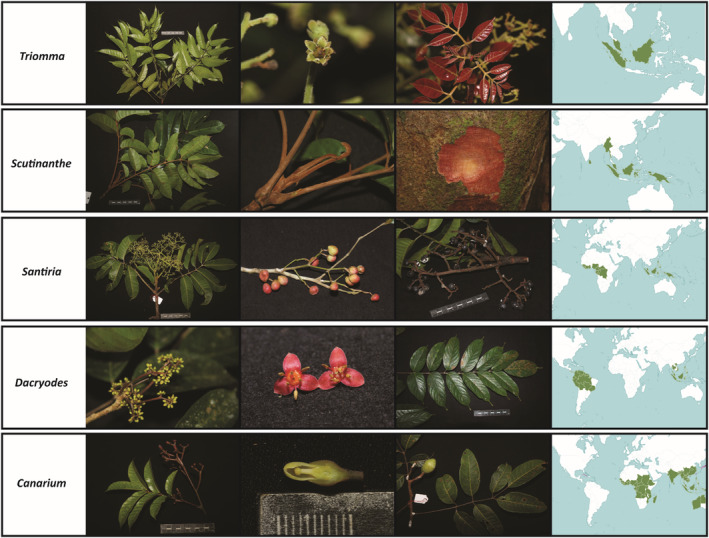
Representatives of each of the five genera (*Triomma*, *Scutinanthe*, *Santiria*, *Dacryodes*, and *Canarium*) from family Burseraceae investigated in this study. Photos by Fabian Brambach. Maps display the native distribution range of each genus colored in dark green. The distribution maps originate from https://powo.science.kew.org.

In this study, 197 specimens representing 20 species from five genera (Figure 1) were collected in the field by our research group and processed in the laboratory (Table S1). A total of 268 sequences from the core barcode loci were used, consisting of 126 *matK* and 142 *rbcL* sequences. Sequences of low quality or for which the BLAST search results matched to taxonomic groups other than Burseraceae were removed from the dataset, and therefore, not included in the analyses (11 *matK* and 12 *rbcL* sequences). The sequencing success rate, excluding contaminated or low quality samples, was higher in *rbcL* than in *matK* (72.1% or 142 samples and 64% or 126 samples, respectively).

As compared to *rbcL*, the *matK* alignment showed a higher number of variable and parsimony‐informative sites, 20.5% and 10.8%, and a total of 9.7% singletons. The *rbcL* sequence alignment contained 16.2% variable sites, 10.8% parsimony‐informative sites, and a total of 9.9% singletons. For both markers aligned, the proportion of variable sites, parsimony‐informative sites, and singletons were 17.8%, 7.6%, and 10.2%, respectively. The G + C content was 44.8% for *rbcL*, 35.6% for *matK*, and 40.6% for the combination of both loci.

### 3.2 Specimen identification using BLAST search

**FIGURE 2:**
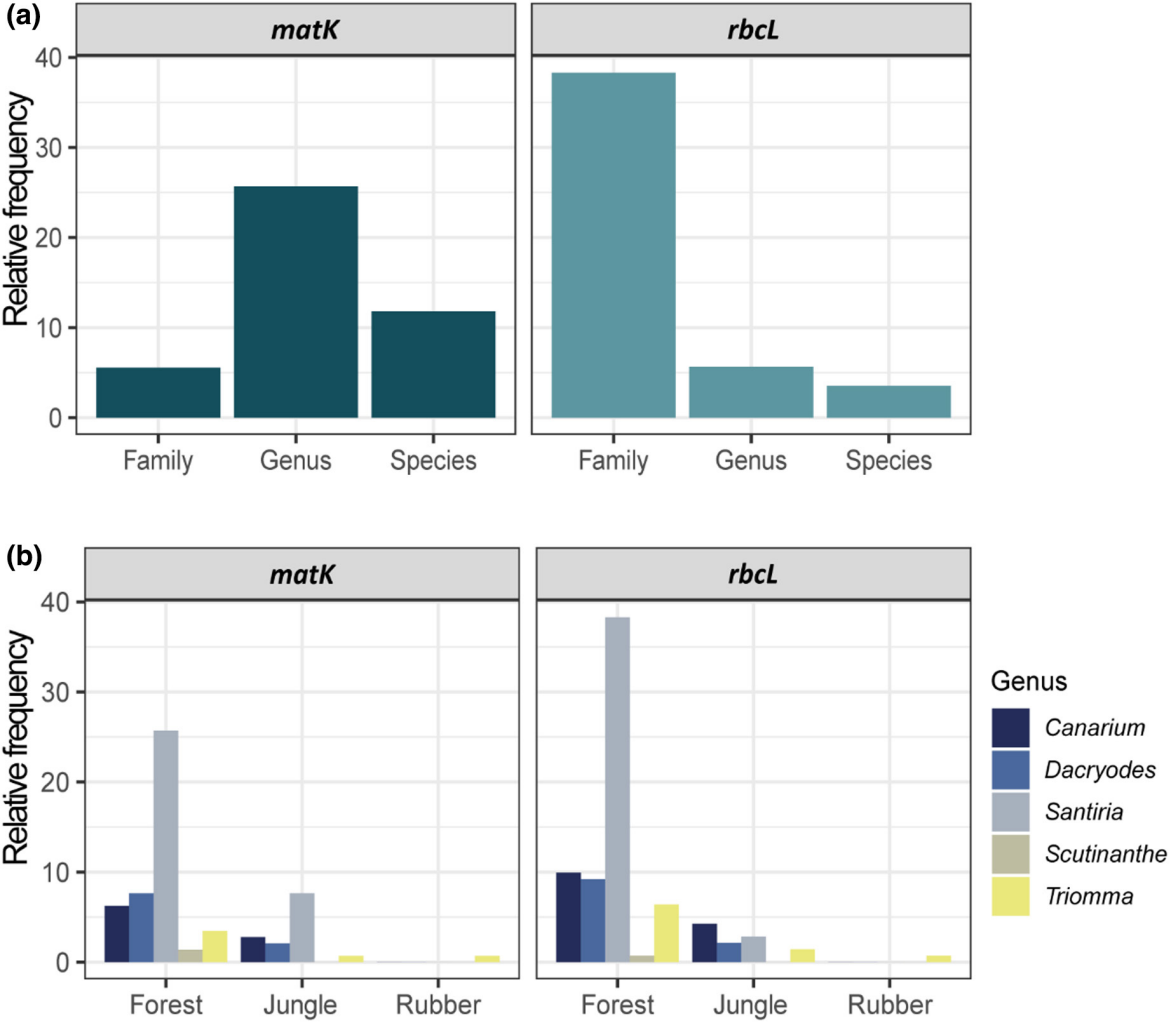
(a) Relative frequency of successful taxonomic assignments at three taxonomic levels (family, genus, and species) evaluated by the correspondence between morphological and molecular identification of Burseraceae specimens using the plant core barcodes *matK* and *rbcL*. (b) Proportion of specimens identified per genus in each land‐use type. No specimens were found in oil palm plots. Jungle corresponds to jungle rubber plots. Relative frequency is represented in percentage.

Molecular identification was conducted using BLAST search of sequences queried against the NCBI database and comparing them with the morphologically identified species. The species names based on the morphological identification were used for every barcode, and the molecular identification success was measured by its congruence with the morphological identification. At the species level, *matK* identified a slightly higher percentage of specimens (28.5%), and *rbcL* had a very low identification rate (6.4%). The highest identification success at the genus level was observed for *matK* (56.25%), followed by *rbcL* (22%). The specimens that could be only identified at family level represented 15.3% for *matK* and 71.6% for *rbcL* (Figure 2a). The most abundant genus identified by DNA barcodes and morphological traits was *Santiria*. Our survey indicated more abundance of Burseraceae specimens in forest plots (Figure 2b).

### 3.3 Genetic distances and barcode gap

As depicted in Table 2, *matK* has the highest interspecific genetic distance with a mean of 0.008, followed by *matK + rbcL* with 0.006, and *rbcL* with 0.004. In this study, the mean interspecific genetic divergence for the single and combined dataset for all samples used is <1%, reinforcing the low genetic variability of the core plant barcode regions to differentiate species in Burseraceae. Besides, *matK* and *rbcL* have 0.003 mean intraspecific divergences, whereas the combined dataset has a mean intraspecific variation of 0.002. The mean intraspecific divergence in *matK* and *matK + rbcL* was significantly lower than the mean interspecific divergence (Wilcoxon rank‐sum test, *p* < .0001). The large range of variation of interspecific genetic divergence, for example, in *matK* ranging from 0% to 3%, indicates that these markers may solve the molecular placement of some of the clades in Burseraceae, for example, for the groups where the interspecific genetic differentiation approaches 3% (Figure S3).

**TABLE 2:** Mean and range values of the interspecific and intraspecific genetic distances estimated using *matK*, *rbcL*, and *matK + rbcL*.

DNA barcodes	Intraspecific divergence		Interspecific divergence	
	Mean	Range	Mean	Range
*matK*	0.003	0.000–0.016	0.008	0.000–0.031
*rbcL*	0.003	0.000–0.012	0.004	0.000–0.020
*matK* + *rbcL*	0.002	0.000–0.007	0.006	0.000–0.023

**FIGURE 3:**
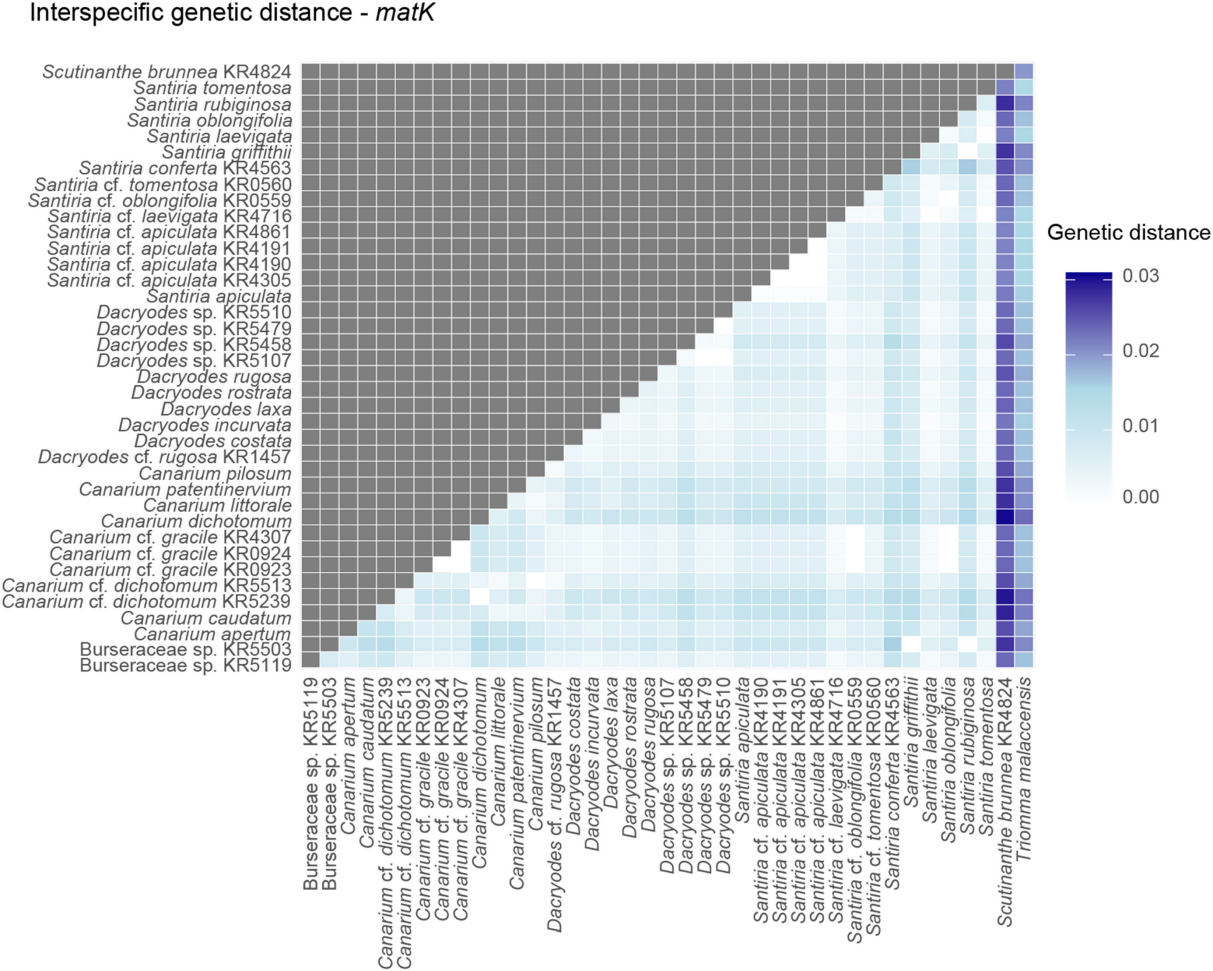
Pairwise genetic distances between species based on *matK* analyzed in this study. Samples with IDs were considered morphologically ambiguous due to unclear morphological identification and were kept separately in the genetic distance estimations to clarify their identification on the molecular basis. Species names without morphological IDs represent the set of sequences grouped by species based on morphological identification.

**FIGURE 4:**
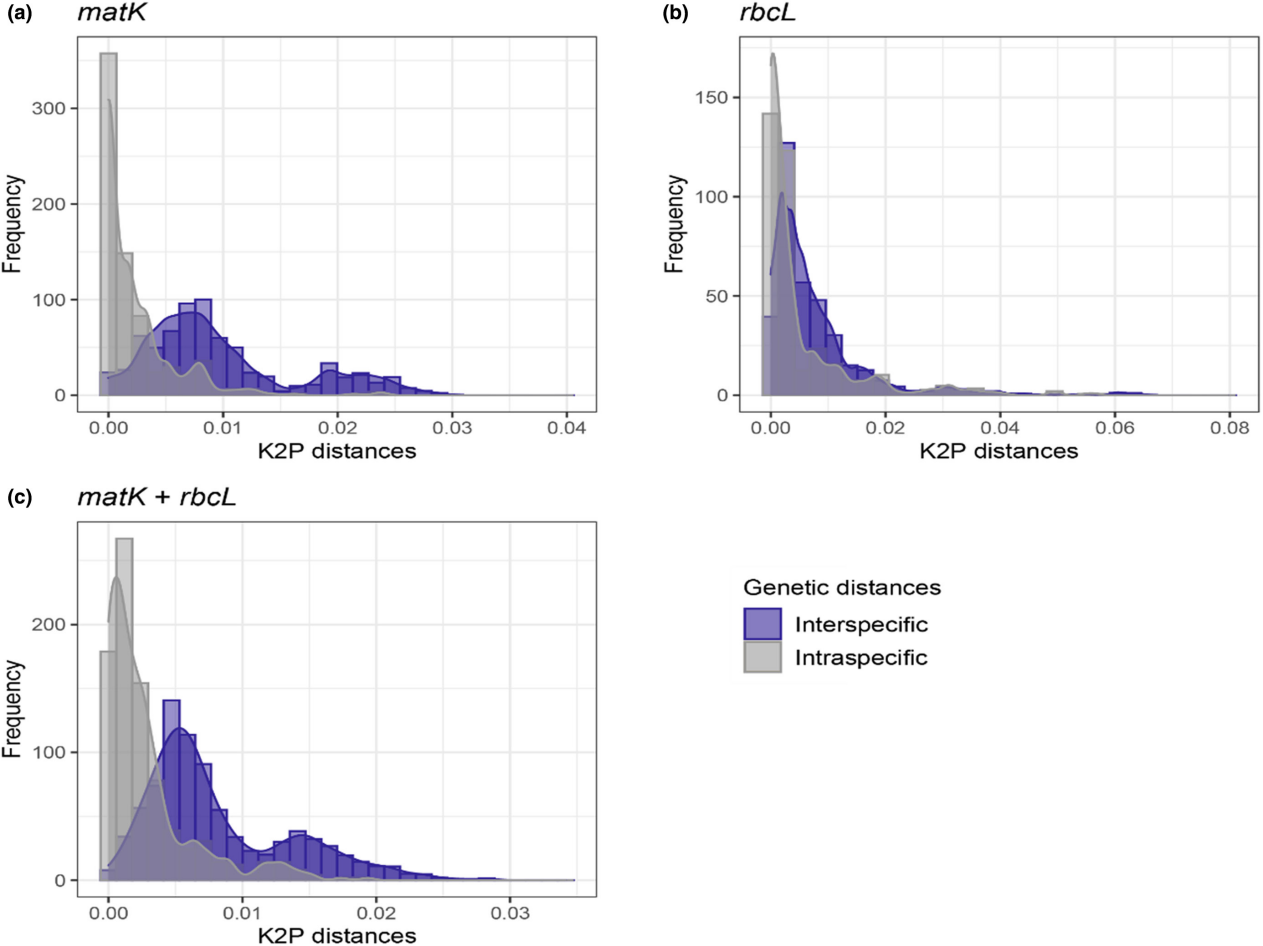
Frequency histogram of K2P inter‐ and intraspecific genetic distances for Burseraceae using *matK*, *rbcL*, and both loci.

Based on the interspecific sequence divergence, *matK* (Figure 3), and the combined dataset, we were able to discriminate 97% of the species pairs (Figure S1); on contrary, *rbcL* discriminated 85% of species pairs as shown in Figure S2. Furthermore, the interspecific genetic distances overlapped with the maximum intraspecific genetic distances for each barcode marker and the combined dataset, and therefore, a lack of a barcode gap between intra‐ and interspecific genetic distances for specimens of family Burseraceae used in this study was observed (Figure 4). However, the mean interspecific genetic divergence was significantly higher than the intraspecific divergence (Wilcoxon rank‐sum test, *p* < .0001; Figure S3).

### 3.4 Molecular assignment using species tree reconstruction

**FIGURE 5:**
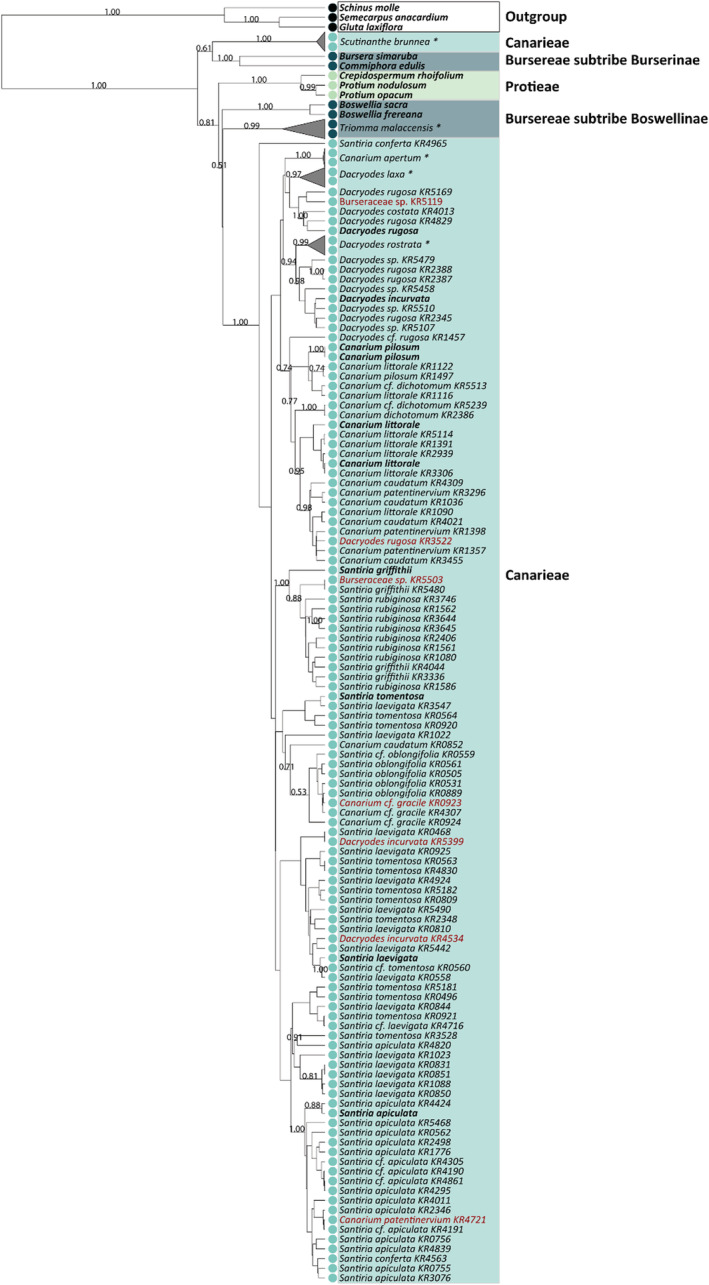
Bayesian inference tree based on *matK* barcode. Nodes are labeled with their respective posterior probabilities, and only nodes with support >0.5 are displayed. Species names with their IDs are displayed on the tips. Colors correspond to tribes of Burseraceae. Tips in bold represent sequences downloaded from NCBI or BOLD. Tips in red highlight sequences of an intruder genus present in the clade. *Monophyletic clades.

The trees based on *matK* and *matK + rbcL* have a higher node support than *rbcL*, in which both recovered five out of 20 of the species as monophyletic (Figure 5 and Figure S3). In addition, five species were monophyletic based on the test for monophyly, *Canarium apertum*, *Dacryodes laxa*, *Dacryodes rostrata*, *Scutinanthe brunnea*, and *Triomma malaccensis* for *matK* (Table 3 and Table S4), whereas *rbcL* identified only one species as monophyletic, *T. malaccensis* (Figure S4).

**TABLE 3:** Test for monophyly results at species level for the Burseraceae samples used in this study based on the *matK* Bayesian inference.

Species	Monophyly	MRCA	Tips	Delta.Tips	Intruders	Intruders
*Boswellia frereana*	Monotypic	NA	1	NA	NA	
*Boswellia sacra*	Monotypic	NA	1	NA	NA	
*Bursera simaruba*	Monotypic	NA	1	NA	NA	
*Canarium apertum*	Yes	210	2	0	0	
*Canarium caudatum*	No	174	5	118	1	*C. patentinervium*
*Canarium dichotomum*	No	177	3	20	0	
*Canarium gracile*	No	243	3	5	1	*S. oblongifolia*
*Canarium littorale*	No	177	9	14	0	
*Canarium patentinervium*	No	174	4	119	2	*D. rugosa*, *C. caudatum*
*Canarium pilosum*	No	178	3	3	2	*C. littorale*, *C. dichotomum*
*Commiphora edulis*	Monotypic	NA	1	NA	NA	
*Crepidospermum rhoifolium*	Monotypic	NA	1	NA	NA	
*Dacryodes costata*	Monotypic	NA	1	NA	NA	
*Dacryodes incurvata*	No	174	3	120	0	
*Dacryodes laxa*	Yes	216	8	0	0	
*Dacryodes rostrata*	Yes	201	2	0	0	
*Dacryodes rugosa*	No	175	8	41	0	
*Protium nodulosum*	Monotypic	NA	1	NA	NA	
*Protium opacum*	Monotypic	NA	1	NA	NA	
*Santiria apiculata*	No	266	18	13	4	*S. tomentosa*, *S. laevigata*, *C. patentinervium*, *S. conferta*
*Santiria conferta*	No	173	2	122	0	
*Santiria griffithii*	No	224	4	9	1	*S. rubiginosa*
*Santiria laevigata*	No	236	17	44	2	*D. incurvata*, *S. tomentosa*
*Santiria oblongifolia*	No	244	5	2	1	*C. gracile*
*Santiria rubiginosa*	No	227	8	2	1	*S. griffithii*
*Santiria tomentosa*	No	236	13	48	0	
*Scutinanthe brunnea*	Yes	154	2	0	0	
*Semecarpus anacardium*	Monotypic	NA	1	NA	NA	
*Triomma malaccensis*	Yes	162	12	0	0	

The *matK* tree placed the two Boswelliinae investigated genera *Triomma* and *Boswellia* in different clades, *Triomma clustering* with *Canarieae* (*Canarium*, *Santiria*, and *Dacryodes*). Tribe Protieae was resolved as monophyletic clade with strong support (PP = 1.0), and subtribe Burserinae was placed in the same clade with *Scutinanthe brunnea* (PP = 0.61) which belongs to tribe Canarieae (Figure 5). The phylogenetic tree constructed using *rbcL* could not resolve even the positions of the tribes and subtribes of the family (Figure S5). Furthermore, the combination of *matK* and *rbcL* did not improve the species identification rate and node support as expected (Figure S4). The phylogenetic tree of *matK + rbcL* did not recover *Santiria*, *Canarium*, and *Dacryodes* as monophyletic. *Boswelliinae* (PP = 0.9), *Protieae* (PP = 0.93), and *Burserinae* (PP = 0.99) were retrieved as monophyletic clades.

## 4 DISCUSSION

### 4.1 Efficiency of the DNA barcodes

One of the main characteristics of an ideal DNA barcode is its recoverability with single primer pairs (CBOL Plant Working group, 2009). High‐sequencing success rate and universality of *rbcL* have been reported by several studies conducted on tropical plant species that achieved sequence recovery ranging from 84% to 95% (e.g., Amandita et al., 2019; Lahaye et al., 2008; Moura et al., 2019). However, in comparison with these studies, a lower recoverability of *rbcL* sequences was achieved (72.1%) in the present study. Low‐sequencing rate of *matK* was reported from a study conducted in a wide range of land plant species by Kress and Erickson (2007); in our study, we were able to sequence 64% of our samples using *matK* primers. Despite the criticism for lack of universal primers for the *matK* region for all land plant species, Lahaye et al. (2008) found 100% sequencing success of *matK* in flowering plants from a biodiversity inventory. Hence, the improvement of primer design may increase this low recovery success of *matK* by increasing the amplification success in angiosperms (Kress & Erickson, 2007).

In addition, evaluation of the suitability of a DNA barcode for species discrimination can be done by employing genetic divergence; therefore, an ideal barcode must have high interspecific and low intraspecific sequence variation (Lahaye et al., 2008). Both barcode markers and the combined dataset used in this study showed significant higher mean interspecific divergence than the mean intraspecific divergence. Similarly, studies conducted on specific plant taxa such as *Otholobium* and *Psoralea* (Bello et al., 2015), Myristicaceae (Newmaster et al., 2008), and Dipterocarpaceae (Moura et al., 2019) obtained congruent results.

Another criterion for the efficacy of a barcode is the pairwise genetic variation for specimens' discrimination. In this regard, *matK* is the most variable region with a mean value of 0.90% and discriminating 97% of the species pairs. Similarly, higher interspecific variability at *matK* was also found in *Otholobium* and *Psoralea* (0.86%; Bello et al., 2015), in Rosaceae (0.90%; Pang et al., 2011), and in the Arctic flora of Canada (1.00%; Saarela et al., 2013). On the contrary, *rbcL* as barcode had a lower performance than *matK* in Burseraceae with an average interspecific divergence of 0.40% and 85% pairwise sequence discrimination. This could be due to the restriction of taxa sampling in this study to the genera of tribe *Canarieae*. For instance, genus *Santiria* and *Dacryodes* have several species with identical *rbcL* sequences of zero interspecific divergence. Furthermore, the discriminatory power of each region may vary depending on the group of plants being studied.

Moreover, the most important function of DNA barcodes is to identify unknown specimens by comparing their sequences with the sequences of already identified species, which are stored in a database (Saarela et al., 2013). Even though 97% of the species pairs in the dataset have been discriminated with the pairwise interspecific divergence using *matK* and *matK + rbcL*, the species identification success rate using BLAST searches against the NCBI database was very low, 39% for *matK + rbcL*, 34% for *matK*, and 6% for *rbcL*. These results are similar to studies that reported the underperformance of DNA barcodes in discriminating at lower taxonomic levels among closely related species (e.g., Bello et al., 2015; Piredda et al., 2011). One of the main reasons for low identification rate of species using BLAST is due to the absence of sequence data of taxa of interest in the NCBI reference library (Amandita et al., 2019). In the present study, 10 (50%) and 6 (30%) of the sampled species had no *matK* and *rbcL* sequences available in the NCBI database, while in BOLD, a total of 18 (90%) of the species used in this study had no available sequences for *matK* and *rbcL*, not including the sequences that have already been made available by our research group previously (Table S2). In addition to that, the low percentage of sequence variability between species, <1% for most of the species, indicates low variation of the core barcodes for species identification in Burseraceae.

From this study, five newly barcoded species have been added to NCBI. Providing newly barcoded species from understudied tropical regions, like Sumatra, to the reference databases will improve the efficiency of molecular species identification. For BOLD, only two species from our pool of sampled specimens had sequences available in the system, excluding the sequences that have previously been made available by our research group. This in turn could contribute to the conservation of biodiversity since accurately identifying the species of interest is the first step toward the identification of hotspots of biodiversity (Kim et al., 2014). Furthermore, the species investigated in this study are underrepresented for other markers. For instance, out the 20 species from Burseraceae investigated in this study, only 20% and 70% of the species have sequences available for *trnH‐psbA* and *trnL‐F*, respectively, and there was only one species with available ITS barcode in NCBI, whereas 50% and 75% of the species have available barcodes of *matK* and *rbcL*, respectively, in NCBI (Table S2). The ITS region has been effectively used for phylogenetic analysis in certain genera of the Burseraceae family (Becerra & Venable, 1999), and it is predicted to be more effective than chloroplast markers due to its higher mutation rate. Nonetheless, the low‐sequencing success rate of this region poses a challenge, making it unsuitable as a universal barcode region (Elbogen, 2012; Gostel et al., 2016).

DNA barcode reference databases can be useful for much more than just instances where morphological identification is difficult (or not available), but in fact, be applied as supplemental tool in addition to the conventional taxonomy in identification and classification of cryptic species (Hartvig et al., 2015; Newmaster & Ragupathy, 2009; Stech et al., 2013). Additionally, it may be incorporated for post hoc ‘molecular’ identifications, where specimens' identification is confirmed afterward by taxonomists with access to the voucher herbarium specimens and molecular species assignments, as conducted in this study. In this study, only five species were recovered as monophyletic and successfully identified based on molecular data. Consequently, DNA barcode identification is dependent on species‐specific level of polymorphism, which varies within the same family, and thus, can be applied as an additional tool for species delimitation.

### 4.2 Species tree assignment

The effectiveness of plant DNA barcoding in identifying species was assessed using the two primary barcode markers, with the percentage of monophyletic species in Burseraceae serving as the basis for evaluation. The phylogenetic trees recovered from *matK*, *rbcL*, and *matK + rbcL* using BI confirmed that *matK* is the most variable region in Burseraceae species sampled in this study. Since *matK* has greater interspecific divergence than intraspecific divergence, its species‐tree had better resolution in which 25% of the species were recovered as monophyletic clades using the BI method. The concatenated data showed similar results, and therefore could not surpass the resolution of *matK* alone. On the contrary, *rbcL* was not variable enough in Burseraceae and could only resolve one species as monophyletic. This is the result of the lower interspecific divergence of *rbcL*, as many species from different genera had identical sequences. For instance, species from genus *Santiria* had identical sequences with species from genus *Dacryodes* and all the sampled species within the genus *Santiria* showed lack of sequence variation. Likewise, Amandita et al. (2019) found that *rbcL* is not sufficiently variable in Burseraceae, in which most species of different genera had identical sequences. This level of performance is considered limited, indicating the need to incorporate additional markers in molecular surveys targeting this plant family.

The phylogenetic trees constructed based on *matK* and *matK* + *rbcL* confirmed the monophyly of Burseraceae and resolved the tribal and subtribal phylogenetic relationships, which mirrors the findings by Clarkson et al. (2002) and Weeks et al. (2005) based on most‐parsimonious trees using *rps16* intron and ETS regions. *Bursereae* is a paraphyletic group, divided into a well‐supported monophyletic group of subtribes *Burserinae* and a paraphyletic subtribe *Boswelliinae*. The *matK* tree placed the two investigated genera of subtribe Boswelliinae, *Triomma* and *Boswellia*, in different clades, *Triomma* clustering with *Canarieae* (*Canarium*, *Santiria*, and *Dacryodes*). Moreover, *Bursera* and *Commiphora*, the two genera of *Burserinae* sampled, were retrieved as one monophyletic group with PP = 1 (Figure 4 and Figure S2), and the previously suspected close association of these two genera is thus substantiated (Clarkson et al., 2002).

Tribe Protieae was resolved as monophyletic clade with strong support (PP = 1.0) and subtribe Burserinae was placed in the same clade with *Scutinanthe brunnea* (PP = 0.61) which belongs to tribe Canarieae (Figure 5). In general, the major clades and relationships among genera and tribes depicted in the *matK* phylogenetic tree are consistent with the results of Clarkson et al. (2002) based on most‐parsimonious trees using *rps16* intron and ETS regions, which recovered the sister relationship between subtribe Boswelliinae and tribe Canarieae similar to this study. However, it is not possible to compare the observed paraphyly of Canarieae in our Bayesian inference, since both studies by Clarkson et al. (2002) and Weeks et al. (2005) lack of *Scutinanthe brunnea* samples. Despite the recommendation of *matK* and *rbcL* by CBOL (CBOL, 2009) as core barcodes for plant identification, our study reinforces the necessity of the development of barcode markers targeting specific groups of plants to increase discriminatory power and accuracy of biodiversity surveys on a molecular basis.

The emergence of high‐throughput sequencing technology (such as Illumina, PacBio, and Oxford Nanopore) has enabled the development of a more comprehensive database of curated barcode sequences from known species. However, the main challenge remains the absence of a complete reference barcode dataset for molecular species assignments (Gostel & Kress, 2022). In light of this, we encourage all initiatives aimed at obtaining DNA barcode sequences to use core barcodes and additional barcode markers to resolve the relationship of closely related taxa, particularly those from tropical species. Each contribution is a valuable step toward filling gaps in the DNA barcode database, promoting its curation, and advancing toward a more complete reference dataset.

## 5 CONCLUSIONS

Our findings show that although *matK* recovered 5 of 20 species as monophyletic clades, it is an useful tool for the identification of selected taxa in such a complex family as Burseraceae. The effectiveness of DNA barcoding in identifying species from the Sumatran tropical rainforest was limited by the availability of reference sequences and species‐specific genetic variability. Nevertheless, *matK* remains a valuable barcode for identifying certain species within Burseraceae. Despite attempts to improve their effectiveness through the combination of chloroplast loci, no significant differences were observed in any of the evaluations conducted. DNA barcoding has the potential to be an effective species identification tool for tropical forests provided that well‐established reference sequence databases are available, and the sequencing success rate is improved. Additional genomic regions could enhance the accuracy of the DNA barcoding method, such as nuclear regions (ITS1 and ETS).

Future studies could evaluate the suitability of DNA barcoding for species delineation and improving the resolution of phylogenetic relationships within Burseraceae by increasing the number of sampled species and genera. Additionally, augmenting reference sequence databases to include missing species and incorporating additional nuclear DNA markers in combination with *matK* may enhance the efficacy of DNA barcoding in this family.

## AUTHOR CONTRIBUTIONS

**Daniel M. Teklemariam:** Conceptualization (lead); data curation (lead); formal analysis (lead); investigation (lead); methodology (lead); software (equal); validation (equal); writing – original draft (lead); writing – review and editing (lead). **Oliver Gailing:** Conceptualization (lead); funding acquisition (lead); investigation (equal); methodology (equal); project administration (supporting); resources (lead); software (supporting); supervision (supporting); validation (supporting); writing – original draft (equal); writing – review and editing (equal). **Fitri Yola Amandita:** Investigation (equal); methodology (equal); writing – review and editing (equal). **Iskandar Z. Siregar:** Conceptualization (equal); funding acquisition (supporting); investigation (supporting); methodology (supporting); project administration (supporting); resources (equal); supervision (supporting); writing – review and editing (equal). **Carina C. M. Moura:** Conceptualization (lead); data curation (lead); formal analysis (lead); funding acquisition (supporting); investigation (lead); methodology (lead); project administration (lead); resources (equal); software (lead); supervision (lead); validation (lead); visualization (lead); writing – original draft (equal); writing – review and editing (equal).

## CONFLICT OF INTEREST STATEMENT

The authors declare no conflict of interest.

### OPEN RESEARCH BADGES

This article has earned an Open Data badge for making publicly available the digitally‐shareable data necessary to reproduce the reported results. The data is available at NCBI Genbank OP587286–OP587371 and OP587372–OP587500.

## Supporting information


Data S1

## Data Availability

NCBI GenBank accession numbers: OP587286–OP587371 and OP587372–OP587500.
