# Supplementary material for: Integrative taxonomy using the plant core DNA barcodes in Sumatra's Burseraceae
Source: Ecol Evol. 2023 Apr 7;13(4):e9935. doi: 10.1002/ece3.9935 (PMC10082172; doi:10.1002/ece3.9935)
Supplement: Supplementary file 1 — Data S1 [file ECE3-13-e9935-s001.docx]

**Supplementary material**


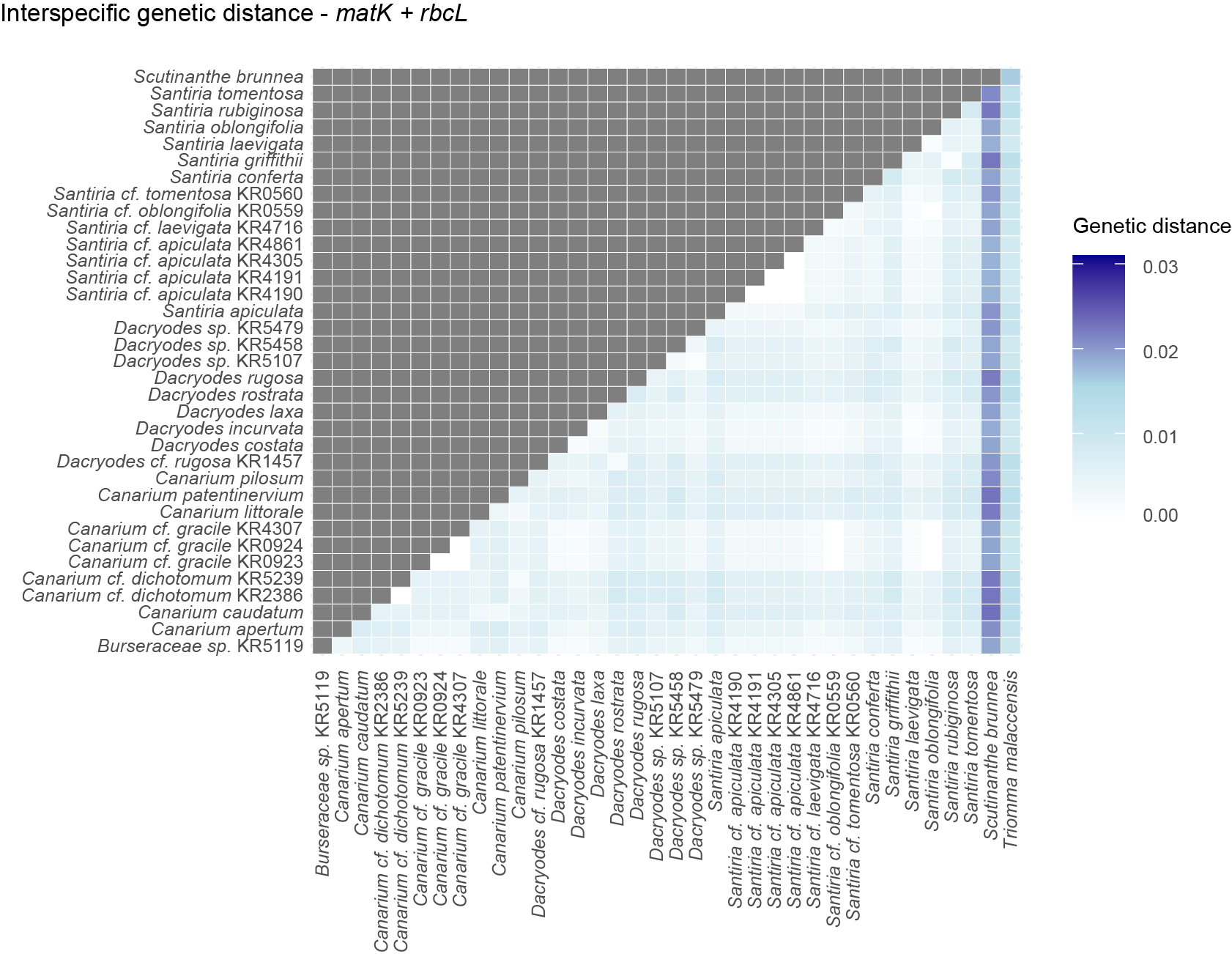


**Figure S1** Pairwise genetic distance between species based on *matK+rbcL*.


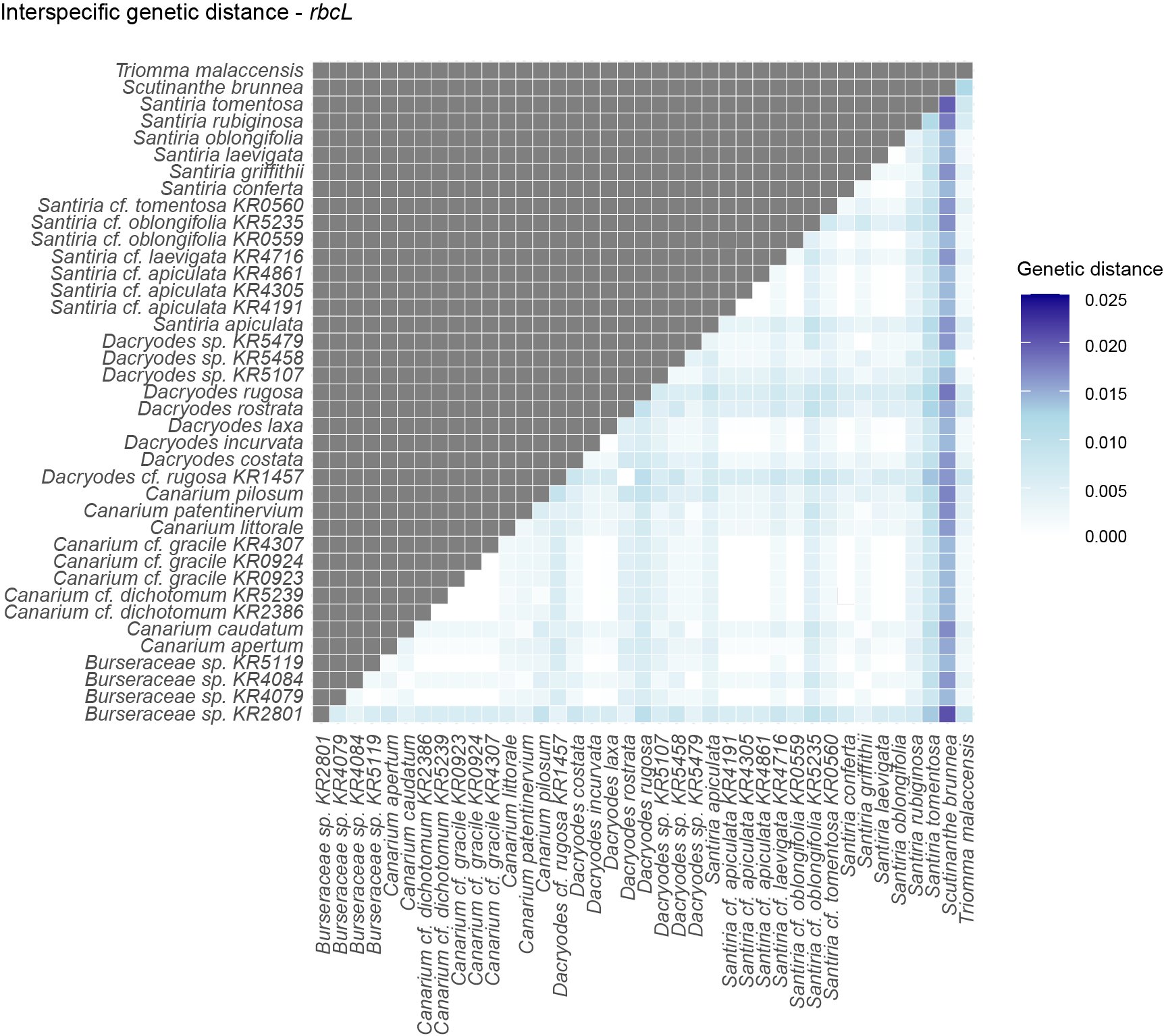


**Figure S2** Pairwise genetic distance between sequences of Burseraceae based on *rbcL*.


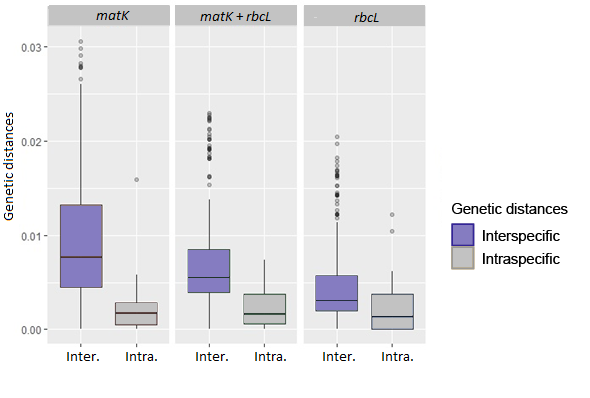


**Figure S3** Box plots show the interspecific divergence (Inter.) and intraspecific divergence (Intra.) of the two DNA markers; *matK, rbcL* and the concatenation of *matK+rbcL* calculated for family Burseraceae. The x-axis shows the divergence type, and the y-axis shows the genetic distances.

**
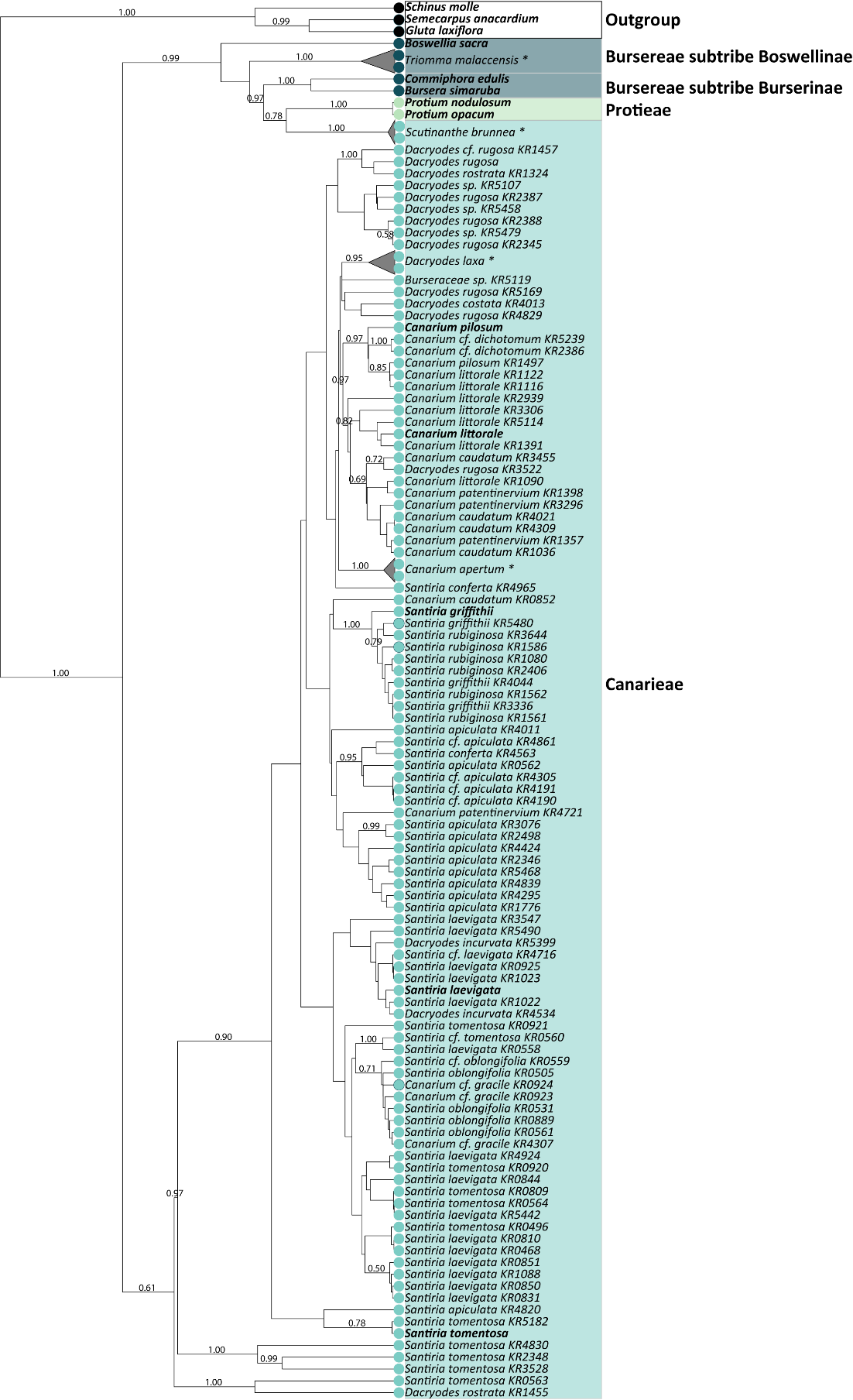
**

**Figure S4** Bayesian Inference tree based on *matK+rbcL* barcode. Nodes are labelled with their respective posterior probabilities which are greater than 0.5 and species names with their IDs are displayed on the tips. In bold, sequences downloaded from the database. * Monophyletic clades collapsed.


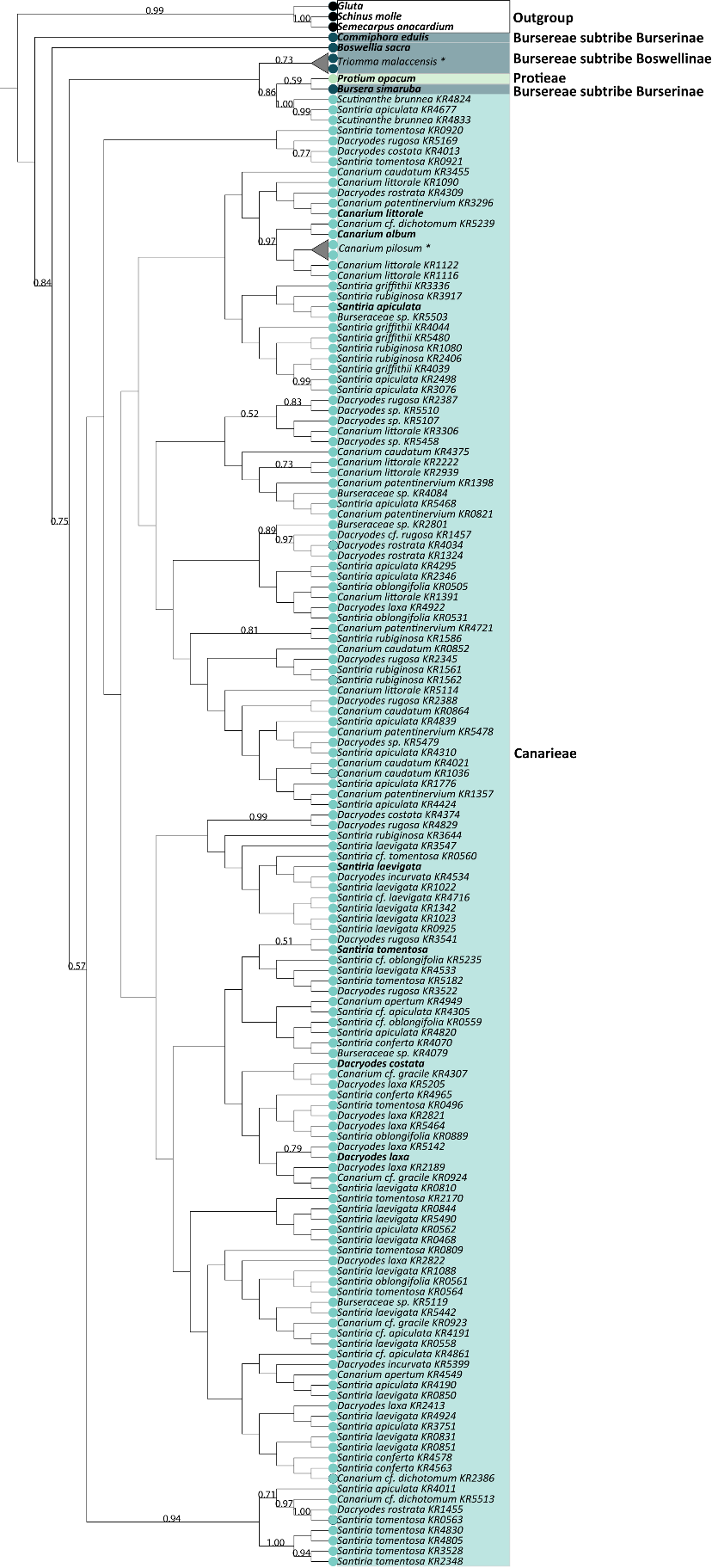


**Figure S5** Bayesian Inference tree based on *rbcL* barcode. Nodes are labeled with their respective posterior probabilities which are greater than 0.5 and species names with their IDs are displayed on the tips. In bold, sequences downloaded from the database. * Monophyletic clades collapsed.

**Table S1** List of samples used in this study with their respective NCBI accession numbers.

| **Sample ID** | **Species** | ***matK* sequences** | ***rbcL* sequences** |
| --- | --- | --- | --- |
| KR4838 | Burseraceae sp. | OP587286 | - |
| KR2801 | Burseraceae sp. | - | OP587372 |
| KR4079 | Burseraceae sp. | - | OP587373 |
| KR4634 | Burseraceae sp. | OP587287 | - |
| KR5106 | Burseraceae sp. | OP587288 | - |
| KR5119 | Burseraceae sp. | OP587289 | OP587375 |
| KR4084 | Burseraceae sp. | - | OP587374 |
| KR5503 | Burseraceae sp. | OP587290 | - |
| KR4549 | *Canarium apertum* | OP587291 | OP587376 |
| KR4949 | *Canarium apertum* | OP587292 | OP587377 |
| KR0852 | *Canarium caudatum* | MH115923.1 | OP587378 |
| KR0864 | *Canarium caudatum* | - | OP587379 |
| KR1036 | *Canarium caudatum* | MH115925.1 | OP587380 |
| KR4021 | *Canarium caudatum* | OP587294 | OP587381 |
| KR4309 | *Canarium caudatum* | OP587295 | OP587382 |
| KR3455 | *Canarium caudatum f. caudatum* | OP587293 | - |
| KR2386 | *Canarium cf. dichotomum* | OP587300 | OP587383 |
| KR5239 | *Canarium cf. dichotomum* | - | OP587384 |
| KR5513 | *Canarium cf. dichotomum* | OP587296 | - |
| KR0923 | *Canarium cf. gracile* | OP587297 | OP587385 |
| KR0924 | *Canarium cf. gracile* | OP587298 | OP587386 |
| KR4307 | *Canarium cf. gracile* | OP587299 | OP587387 |
| KR1090 | *Canarium littorale* | MH115938.1 | OP587388 |
| KR1116 | *Canarium littorale* | MH115941.1 | OP587389 |
| KR1122 | *Canarium littorale* | OP587301 | OP587390 |
| KR1391 | *Canarium littorale* | OP587302 | OP587391 |
| KR2222 | *Canarium littorale* | - | OP587392 |
| KR2939 | *Canarium littorale* | OP587303 | OP587393 |
| KR3306 | *Canarium littorale* | - | OP587394 |
| KR5114 | *Canarium littorale* | OP587304 | OP587395 |
| KR0821 | *Canarium patentinervium* | - | OP587396 |
| KR3296 | *Canarium patentinervium* | OP587307 | OP587399 |
| KR1357 | *Canarium patentinervium* | OP587305 | OP587397 |
| KR1398 | *Canarium patentinervium* | OP587306 | OP587398 |
| KR4721 | *Canarium patentinervium* | OP587308 | - |
| KR5478 | *Canarium patentinervium* | - | OP587400 |
| KR1413 | *Canarium pilosum* | - | OP587401 |
| KR1497 | *Canarium pilosum* | OP587309 | OP587402 |
| KR1457 | *Dacryodes cf. rugosa* | OP587310 | OP587403 |
| KR4013 | *Dacryodes costata* | OP587311 | OP587404 |
| KR4374 | *Dacryodes costata* | OP587312 | OP587405 |
| KR4534 | *Dacryodes incurvata* | OP587312 | OP587406 |
| KR5399 | *Dacryodes incurvata* | OP587313 | OP587407 |
| KR2189 | *Dacryodes laxa* | OP587314 | OP587408 |
| KR2413 | *Dacryodes laxa* | MH115926.1 | OP587409 |
| KR2821 | *Dacryodes laxa* | - | OP587410 |
| KR2822 | *Dacryodes laxa* | OP587315 | OP587411 |
| KR4922 | *Dacryodes laxa* | OP587316 | OP587412 |
| KR5142 | *Dacryodes laxa* | OP587317 | OP587413 |
| KR5205 | *Dacryodes laxa* | - | OP587414 |
| KR5464 | *Dacryodes laxa* | OP587318 | OP587415 |
| KR1324 | *Dacryodes rostrata* | MH115930.1 | OP587416 |
| KR1455 | *Dacryodes rostrata* | MH115946.1 | - |
| KR4034 | *Dacryodes rostrata* | - | OP587417 |
| KR2345 | *Dacryodes rugosa* | - | OP587418 |
| KR2387 | *Dacryodes rugosa* | MH115911.1 | OP587419 |
| KR2388 | *Dacryodes rugosa* | OP587319 | OP587420 |
| KR3522 | *Dacryodes rugosa* | OP587320 | OP587421 |
| KR3541 | *Dacryodes rugosa* | - | OP587422 |
| KR4829 | *Dacryodes rugosa* | OP587321 | OP587423 |
| KR5169 | *Dacryodes rugosa* | OP587322 | - |
| KR5510 | *Dacryodes sp* | OP587325 | - |
| KR5458 | *Dacryodes sp.* | - | OP587425 |
| KR5479 | *Dacryodes sp.* | OP587324 | OP587426 |
| KR5107 | *Dacryodes* sp. | OP587323 | OP587424 |
| KR0562 | *Santiria apiculata* | MH115913.1 | OP587427 |
| KR0755 | *Santiria apiculata* | MH115908.1 | - |
| KR0756 | *Santiria apiculata* | OP587326 | - |
| KR1776 | *Santiria apiculata* | MH115942.1 | OP587428 |
| KR2346 | *Santiria apiculata* | MH115927.1 | OP587429 |
| KR2498 | *Santiria apiculata* | OP587327 | OP587430 |
| KR3076 | *Santiria apiculata* | OP587328 | OP587431 |
| KR3751 | *Santiria apiculata* | - | OP587432 |
| KR4295 | *Santiria apiculata* | OP587330 | OP587434 |
| KR4677 | *Santiria apiculata* | - | OP587437 |
| KR4820 | *Santiria apiculata* | OP587331 | - |
| KR4839 | *Santiria apiculata* | OP587332 | OP587438 |
| KR5468 | *Santiria apiculata* | - | OP587439 |
| KR4310 | *Santiria apiculata* | - | OP587435 |
| KR4424 | *Santiria apiculata* | - | OP587436 |
| KR4011 | *Santiria apiculate* | OP587329 | - |
| KR4190 | *Santiria cf. apiculata* | OP587334 | OP587433 |
| KR4191 | *Santiria cf. apiculata* | OP587335 | OP587445 |
| KR4305 | *Santiria cf. apiculata* | OP587333 | OP587440 |
| KR4861 | *Santiria cf. apiculata* | OP587336 | OP587441 |
| KR4716 | *Santiria cf. laevigata* | OP587337 | OP587442 |
| KR0559 | *Santiria cf. oblongifolia* | OP587338 | OP587443 |
| KR0560 | *Santiria cf. tomentosa* | MH115917.1 | OP587444 |
| KR4578 | *Santiria conferta* | - | OP587448 |
| KR4070 | *Santiria conferta* | OP587446 | - |
| KR4563 | *Santiria conferta* | - | OP587447 |
| KR4965 | *Santiria conferta* | OP587339 | - |
| KR3336 | *Santiria griffithii* | OP587340 | OP587449 |
| KR4039 | *Santiria griffithii* | - | OP587450 |
| KR4044 | *Santiria griffithii* | OP587341 | OP587451 |
| KR5480 | *Santiria griffithii* | OP587342 | - |
| KR0468 | *Santiria laevigata* | MH115916.1 | OP587452 |
| KR0558 | *Santiria laevigata* | MH115910.1 | OP587453 |
| KR0844 | *Santiria laevigata* | - | OP587456 |
| KR0851 | *Santiria laevigata* | OP587346 | OP587458 |
| KR0925 | *Santiria laevigata* | MH115921.1 | OP587459 |
| KR1022 | *Santiria laevigata* | MH115922.1 | OP587460 |
| KR1023 | *Santiria laevigata* | MH115929.1 | OP587461 |
| KR5442 | *Santiria laevigata* | OP587350 | OP587467 |
| KR5490 | *Santiria laevigata* | OP587351 | OP587468 |
| KR0810 | *Santiria laevigata* | OP587343 | OP587454 |
| KR0831 | *Santiria laevigata* | OP587344 | OP587455 |
| KR0850 | *Santiria laevigata* | OP587345 | OP587457 |
| KR1088 | *Santiria laevigata* | OP587347 | OP587462 |
| KR1342 | *Santiria laevigata* | - | OP587463 |
| KR3547 | *Santiria laevigata* | OP587348 | OP587464 |
| KR4533 | *Santiria laevigata* | - | OP587465 |
| KR4924 | *Santiria laevigata* | OP587349 | OP587466 |
| KR0505 | *Santiria oblongifolia* | MH115920.1 | OP587469 |
| KR0531 | *Santiria oblongifolia* | OP587351 | OP587470 |
| KR0561 | *Santiria oblongifolia* | MH115915.1 | OP587471 |
| KR0889 | *Santiria oblongifolia* | MH115919.1 | OP587472 |
| KR1586 | *Santiria rubiginosa* | OP587355 | OP587476 |
| KR3644 | *Santiria rubiginosa* | OP587357 | OP587478 |
| KR3645 | *Santiria rubiginosa* | OP587358 | - |
| KR3746 | *Santiria rubiginosa* | OP587359 | - |
| KR1080 | *Santiria rubiginosa* | MH115947.1 | OP587473 |
| KR1561 | *Santiria rubiginosa* | OP587353 | OP587474 |
| KR1562 | *Santiria rubiginosa* | OP587354 | OP587475 |
| KR2406 | *Santiria rubiginosa* | OP587356 | OP587477 |
| KR3917 | *Santiria rubiginosa* | - | OP587479 |
| KR0496 | *Santiria tomentosa* | MH115940.1 | - |
| KR0563 | *Santiria tomentosa* | MH115924.1 | - |
| KR0564 | *Santiria tomentosa* | MH115914.1 | - |
| KR0809 | *Santiria tomentosa* | MH115939.1 | - |
| KR0920 | *Santiria tomentosa* | MH115937.1 | OP587480 |
| KR0921 | *Santiria tomentosa* | MH115945.1 | OP587481 |
| KR2170 | *Santiria tomentosa* | - | OP587482 |
| KR2348 | *Santiria tomentosa* | MH115935.1 | OP587483 |
| KR3528 | *Santiria tomentosa* | OP587360 | OP587484 |
| KR4805 | *Santiria tomentosa* | - | OP587485 |
| KR4830 | *Santiria tomentosa* | OP587361 | OP587486 |
| KR5181 | *Santiria tomentosa* | OP587362 | OP587487 |
| KR5182 | *Santiria tomentosa* | OP587363 | - |
| KR4824 | *Scutinanthe brunnea* | OP587364 | OP587488 |
| KR4833 | *Scutinanthe brunnea* | OP587365 | - |
| KR3671 | *Triomma malaccensis* | - | OP587491 |
| KR4096 | *Triomma malaccensis* | - | OP587492 |
| KR4308 | *Triomma malaccensis* | - | OP587494 |
| KR4555 | *Triomma malaccensis* | - | OP587495 |
| KR4634 | *Triomma malaccensis* | - | OP587496 |
| KR5106 | *Triomma malaccensis* | - | OP587497 |
| KR5121 | *Triomma malaccensis* | OP587369 | OP587498 |
| KR5447 | *Triomma malaccensis* | OP587370 | OP587499 |
| KR5467 | *Triomma malaccensis* | OP587371 | OP587500 |
| KR1397 | *Triomma malaccensis* | OP587366 | OP587489 |
| KR2812 | *Triomma malaccensis* | OP587367 | OP587490 |
| KR4118 | *Triomma malaccensis* | OP587368 | OP587493 |

**Table S2** List of species from Burseraceae sampled in this study and the availability of several DNA barcodes reference sequences for these species in NCBI.

|  | ***BOLD*** | | ***NCBI*** | | | | |
| --- | --- | --- | --- | --- | --- | --- | --- |
| **Species** | ***matK*** | ***rbcL*** | ***matK*** | ***rbcL*** | **ITS** | ***trnH- psbA*** | ***trnL-F*** |
| *Canarium apertum* H.J.Lam | No | No | No | No | No | No | No |
| *Canarium caudatum* King | Yes* | Yes* | Yes* | Yes* | No | No | No |
| *Canarium dichotomum*  Miq | Yes* | Yes* | No | Yes* | No | No | No |
| *Canarium littorale* Blume | Yes* | Yes* | Yes | Yes | No | Yes | Yes |
| *Canarium patentinervium* Miq. | No | Yes* | No | Yes | No | No | No |
| *Canarium pilosum* A.W.Benn | No | Yes* | Yes | Yes | No | Yes | Yes |
| *Dacryodes costata* A.W.Benn | No | No | Yes | Yes | No | No | Yes |
| *Dacryodes incurvata* (Engl.) H.J.Lam | Yes | Yes | No | No | No | No | No |
| *Dacryodes laxa* A.W.Benn | Yes* | Yes* | Yes | Yes | No | No | Yes |
| *Dacryodes rostrata* Blume | Yes* | Yes* | Yes* | Yes | No | No | Yes |
| *Dacryodes rugosa* Blume | Yes* | Yes* | Yes | Yes | No | No | Yes |
| *Santiria apiculata* A.W.Benn | Yes* | Yes* | Yes | Yes | No | No | Yes |
| *Santiria conferta* A.W.Benn. | Yes | Yes | No | Yes | No | No | Yes |
| *Santiria griffithii* Hook.f. | Yes* | Yes* | Yes | Yes | Yes | Yes | No |
| *Santiria laevigata* Blume | Yes* | Yes* | Yes | Yes | No | No | No |
| *Santiria oblongifolia* Blume | Yes* | Yes* | Yes* | Yes | No | No | Yes |
| *Santiria rubiginosa* Blume | Yes* | Yes* | Yes* | Yes | No | No | Yes |
| *Santiria tomentosa* Blume | Yes* | Yes* | Yes | Yes | No | No | Yes |
| *Scutinanthe brunnea* Thwaites | No | No | No | No | No | No | No |
| *Triomma malaccensis* Hook.f. | No | No | Yes | Yes | No | No | Yes |

Yes – Sequence of the species is available in NCBI or BOLD.

No - Sequence is not available in NCBI nor BOLD.

*Sequence has been submitted to NCBI or BOLD previously by our research group.

**Table S3** List of sequences downloaded from NCBI and BOLD.

| **Species** | **Sample ID** | **Accession No** | **Database** | **Barcode** |
| --- | --- | --- | --- | --- |
| *Boswellia frereana* Birdw | AY594461 | AY594461 | NCBI | *matK* |
| *Boswellia sacra* Flück | US Gostel277 | MH748929 | NCBI | *matK* |
| *Canarium littorale* Blume | Gp-400 | MH332598 | NCBI | *matK* |
| *Canarium pilosum* A.W.Benn | BT0095963737 | KJ708857 | NCBI | *matK* |
| *Commiphora edulis* Engl. | JF270711 | RBN341 | NCBI | *matK* |
| *Crepidospermum rhoifolium* Triana &Planch | DC40749 | SBIO013-17 | BOLD | *matK* |
| *Dacryodes laxa* A.W.Benn | 22-1106 | MF418864 | NCBI | *matK* |
| *Dacryodes rugosa* Blume | BT0095963724 | KJ708878 | NCBI | *matK* |
| *Gluta laxiflora* Ridl | 20-5481 | BABRU3790-15 | BOLD | *matK* |
| *Santiria griffithii* Hook.f. | BT0095963784 | KJ709068 | NCBI | *matK* |
| *Santiria laevigata* Blume | BT0070234372 | KJ709069 | NCBI | *matK* |
| *Santiria tomentosa* Blume | gp-376 | MH332584 | NCBI | *matK* |
| *Schinus molle* L. | HOSAM00054 | HOSAM054-10 | BOLD | *matK* |
| *Semecarpus anacardium* L.f. | SRM000248A | TRM079-12 | NCBI | *matK* |
| *Triomma malaccensis* Hook.f. | BT0070234241 | KJ709125 | NCBI | *matK* |
| *Protium nodulosum* Swart | DC42094 | SBIO068-17 | BOLD | *matK*-*rbcL* |
| *Protium opacum* Swart | DC42248 | SBIO089-17 | BOLD | *matK*-*rbcL* |
| *Beiselia mexicana* Forman | GU246019 | GBVR4492-13 | BOLD | *rbcL* |
| *Bursera biflora* Standl | Gu246023.1 | GBVR4496-13 | BOLD | *rbcL* |
| *Dacryodes rostrata* Blume | D14288 | KT698521 | NCBI | *rbcL* |
| *Gluta laxiflora* Ridl. | 01-3865 | BABRU1061-14 | BOLD | *rbcL* |
| *Santiria apiculata* A.W.Benn | gp351 | MH332435 | NCBI | *rbcL* |
| *Schinus molle* L. | MVdB0046 | JX572951 | NCBI | *rbcL* |
| *Semecarpus anacardium* L.f. | SRM00403A | API103-12 | BOLD | *rbcL* |
| *Triomma malaccensis* Hook.f. | BT0070234241 | KJ594922.1 | NCBI | *rbcL* |

**Table S4** Test monophyly results at genus level for the Burseraceae samples used in this study.

| **Species** | **Monophyly** | **MRCA** | **Tips** | **Delta.Tips** | **Intruders** | **Intruders** |
| --- | --- | --- | --- | --- | --- | --- |
| *Boswellia* | Yes | 160 | 2 | 0 | 0 |  |
| *Bursera* | Monotypic | NA | 1 | NA | NA |  |
| *Canarium* | No | 174 | 29 | 94 | 1 | *Dacryodes* |
| *Commiphora* | Monotypic | NA | 1 | NA | NA |  |
| *Crepidospermum* | Monotypic | NA | 1 | NA | NA |  |
| *Dacryodes* | No | 174 | 26 | 97 | 2 | *Canarium* |
| *Protium* | Yes | 158 | 2 | 0 | 0 |  |
| *Santiria* | No | 173 | 67 | 57 | 3 | *Dacryodes, Canarium* |
| *Scutinanthe* | Yes | 154 | 2 | 0 | 0 |  |
| *Semecarpus* | Monotypic | NA | 1 | NA | NA |  |
| *Triomma* | Yes | 162 | 12 | 0 | 0 |  |
